# Pain spectrum in immune checkpoint inhibitor-related adverse events: evolution, characteristics and management challenges based on bibliometrics

**DOI:** 10.1007/s00520-026-10440-4

**Published:** 2026-02-25

**Authors:** Qiongqian Li, Shunrong Zhang, Tongze Cai, Juanmei Mo, Huang Tang, Zhiyong Yu, Xiaoming Zheng, Jianlong Zhou, Guodong Huang

**Affiliations:** 1 Guangxi International Zhuang Medicine Hospital, Affiliated to Guangxi University of Chinese Medicine, Nanning, China; 2 Ruikang Hospital, Affiliated to Guangxi University of Chinese Medicine, Nanning, China

**Keywords:** Bibliometric analysis, Cancer, Immune checkpoint inhibitors, Adverse events, Pain

## Abstract

**Background:**

Immune checkpoint inhibitors (ICIs) have transformed cancer treatment, driving increased research into immune-related adverse events (irAEs), including pain. ICI-induced pain differs from traditional cancer pain or pain caused by chemotherapy or radiotherapy in its mechanisms, manifestations, and management. It often necessitates ICI dose reduction, potentially compromising efficacy, and requires careful balancing of immunosuppression and analgesia. Although glucocorticoids are first line for inflammatory pain, their long-term or high-dose use can attenuate ICI effectiveness, increase infection risk, and lead to pain recurrence after withdrawal—making “steroid-sparing strategies” a central clinical challenge. However, bibliometric studies in this area remain scarce. This analysis of 484 publications examines research trends, advances, characteristics, and management challenges of the pain spectrum in ICI-related irAEs.

**Methods:**

A systematic literature search was performed in the Web of Science Core Collection (WoSCC), yielding 484 relevant papers. Visualization and analysis were conducted using VOSviewer and CiteSpace. Collaboration networks, keyword co-occurrence, citation relationships, and citation burst detection were analyzed to identify research structures and current focal points.

**Results:**

Oncology forms the core discipline, with U.S. institutions playing a leading role. International collaboration is vital for advancing the field. Current research focuses on characterizing pain phenotypes across different ICIs and elucidating their potential immunological mechanisms. A major clinical challenge lies in early identification and differentiation of ICI-related pain from tumor- or treatment-related pain. Equally important is balancing rapid irAE control with avoiding the negative impact of prolonged steroid use on pain outcomes. Developing “steroid-sparing strategies” and establishing predictive biomarker systems are essential for achieving precision pain management.

**Conclusion:**

This bibliometric study maps the knowledge landscape and developmental trends of pain in ICI-related adverse events, underscores the need for interdisciplinary collaboration, and highlights future directions for overcoming clinical challenges and advancing individualized, precision-based treatment strategies.

**Supplementary information:**

The online version contains supplementary material available at 10.1007/s00520-026-10440-4.

## Introduction

Immune checkpoint inhibitors (ICIs)—including antibodies targeting programmed cell death-1 (PD-1), programmed death-ligand 1 (PD-L1), and cytotoxic T-lymphocyte-associated protein 4 (CTLA-4)—have revolutionized the treatment of advanced cancers by mitigating immunosuppression within the tumor microenvironment, thereby substantially improving patient survival [1]. However, this potentiated anti-tumor immune response acts as a “double-edged sword,” frequently inducing immune-related adverse events (irAEs) that involve multiple organ systems such as the skin, gastrointestinal tract, endocrine glands, liver, and lungs. These irAEs pose considerable challenges to patients’ quality of life and treatment adherence [2].

Among the diverse spectrum of irAEs, pain represents a particularly prominent and clinically complex manifestation. The term “pain spectrum” in this context refers to a constellation of symptoms driven by immune-mediated tissue inflammation and injury resulting from ICI therapy, with pain serving as the primary clinical presentation. This spectrum encompasses a heterogeneous array of pain phenotypes, including but not limited to neuropathic, musculoskeletal, visceral, headache, and diffuse pain [3–5]. Its heterogeneity, multisystem involvement, and diagnostic complexity necessitate multidisciplinary collaboration for effective management. Clinical observations and retrospective studies indicate that painful irAEs exhibit a high incidence, are often associated with significant functional impairment and emotional distress, and markedly compromise patients’ quality of life [6]. In severe cases, they can be life-threatening or lead to permanent disability, frequently requiring interruption or dose modification of ICI therapy [7–10]. A key diagnostic challenge lies in accurately attributing the etiology of pain, which may arise from direct immune attack on specific tissues by ICIs, occur as a concomitant symptom of other irAEs (e.g., pancreatitis, pneumonitis, or colitis), or be related to the underlying malignancy or other treatments such as radiotherapy and chemotherapy [11, 12]. This etiological complexity complicates clinical diagnosis, assessment, and management, and there remains a lack of standardized, evidence-based guidelines for pain management in this setting.

Despite the growing clinical recognition of ICI-related pain, most existing studies have been limited to reports on specific pain types or small cohort analyses. A systematic overview of the overall research landscape, knowledge structure, and evolutionary trajectory in this field is currently lacking. Bibliometrics offers a robust analytical framework to address this gap [13]. Through quantitative analysis and visualization of scientific literature, this methodology can objectively and systematically delineate the research structure, dynamic evolution, focal points, and disciplinary impact within a given field [14]. For the emerging and inherently multidisciplinary domain of ICI-related pain—spanning oncology, immunology, neuroscience, rheumatology, and pain medicine—a bibliometric investigation is particularly warranted. By synthesizing the current research status and forecasting future trends regarding the pain spectrum in ICI-associated adverse events, this study aims to provide valuable reference and insight for researchers in the field.

## Materials and methods

### Data collection and search strategies

Literature published in English was retrieved from the Science Citation Index Expanded (SCI-E), a sub-database within the Web of Science Core Collection (WoSCC). While other databases such as PubMed, Scopus, and Google Scholar are available, WoSCC offers several distinct advantages for bibliometric analysis [15]. As one of the world’s largest and most authoritative scientific citation databases, WoSCC provides extensive coverage of multidisciplinary academic literature, including biomedicine. It is renowned for its rigorous selection criteria and quality control, ensuring the inclusion of high-impact and authoritative publications. Furthermore, WoSCC employs standardized metadata and indexing, which facilitates systematic data retrieval and analysis. Among its sub-databases, SCI-E is widely regarded as the preferred source for bibliometric studies due to its broad acceptance, comprehensive coverage, and high practicality [16, 17]. Consequently, we selected SCI-E within WoSCC as the sole data source for this study.

The specific search strategy was designed to capture literature on pain associated with ICI therapy and was executed on August 19, 2025. The search query combined terms related to immune checkpoint inhibitors, pain, and adverse events as follows: (TS = “immune checkpoint inhibitor*” OR “anti-PD-1” OR “anti-PD-L1” OR “anti-CTLA-4” OR “ipilimumab” OR “nivolumab” OR “pembrolizumab” OR “atezolizumab” OR “durvalumab” OR “avelumab” OR “cemiplimab” OR “tislelizumab” OR “toripalimab” OR “ICI”) AND (TS = “pain” OR “arthralgia” OR “myalgia” OR “neuralgia” OR “neuropathic pain” OR “abdominal pain” OR “chest pain” OR “bone pain” OR “headache”) AND (TS = “adverse event*” OR “irAE*” OR toxicity).

**Fig. 1:**
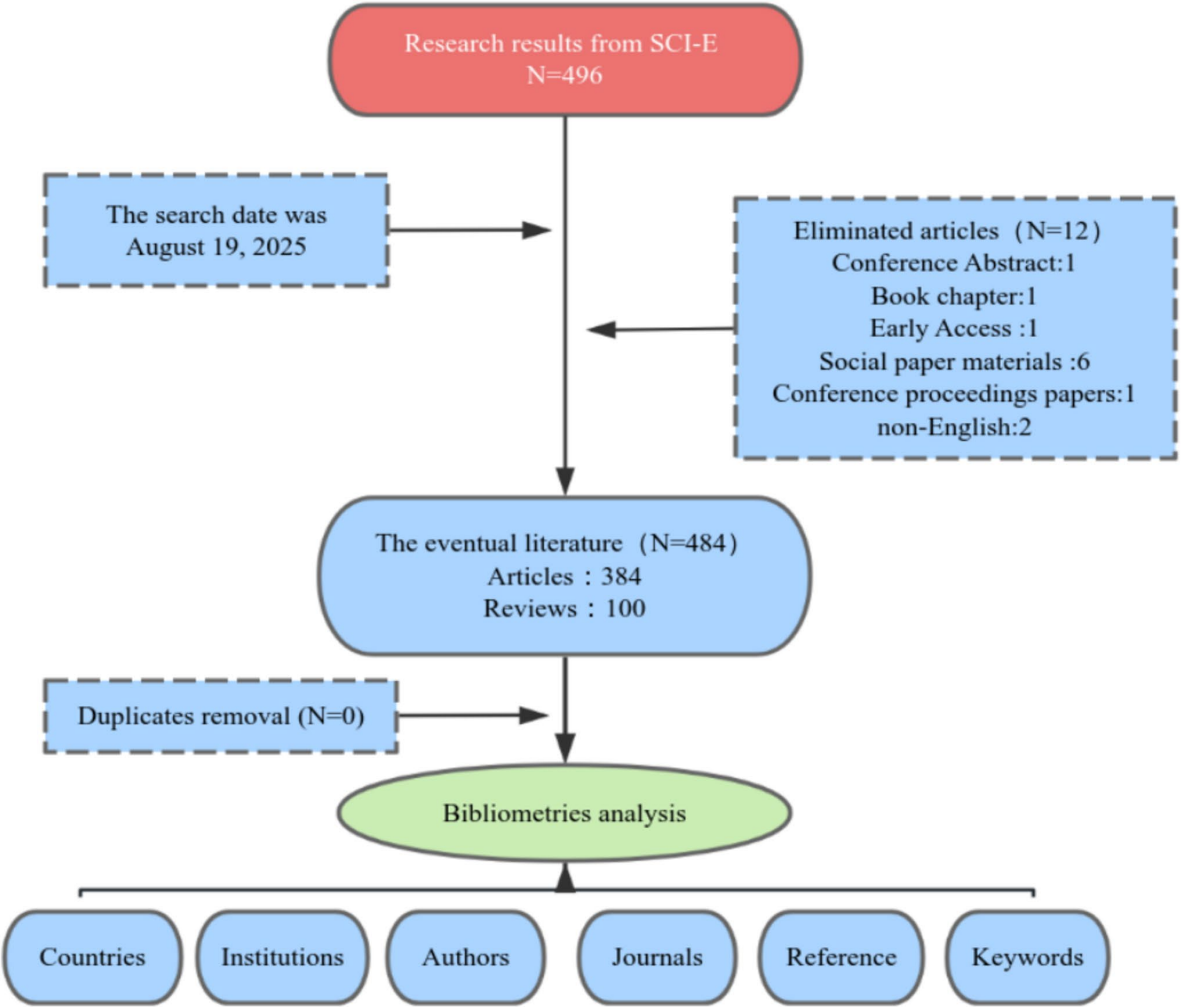
Flowchart of literature search and selection process. The diagram outlines the systematic retrieval and screening procedure for studies on pain spectrum in immune checkpoint inhibitor-related adverse events, from initial identification in the Web of Science Core Collection to final inclusion of 484 articles

To ensure the accuracy and reproducibility of the search results and to minimize potential bias introduced by daily database updates, two researchers independently performed the literature retrieval within a single day. The retrieved records included complete metadata such as title, keywords, abstract, authors, affiliations, and references. All data were exported and stored in plain text format for subsequent analysis. A flowchart detailing the search and screening process is provided in Fig. 1.

### Inclusion and exclusion criteria

Following an initial search, 496 records were identified for further assessment. The inclusion criteria encompassed studies directly relevant to the research topic, with document types limited to original research articles and reviews published in English. Exclusion criteria were applied to remove records deemed irrelevant to the subject matter, as well as non-article publication types such as conference abstracts, book chapters, editorials, letters, and proceedings, in addition to publications with incomplete content or duplicates.

Subsequently, a detailed manual screening based on these criteria was conducted independently by two researchers to ensure the accuracy and validity of the selection process. Any disagreements regarding the eligibility of specific records were documented and resolved through a joint re-evaluation of the original source. In cases where consensus could not be reached, a third senior researcher was consulted to make a final determination.

Thereafter, 484 articles were selected for in-depth bibliometric analysis. The final corpus comprised 384 original research articles and 100 review papers. A complete list of the included studies is provided in Supplementary Table S1.

### Data analysis and visualization

Bibliometric analysis and visualization were performed using VOSviewer (version 1.6.18.0) and CiteSpace (version 6.4.R1) software. Annual publication trends and total citation counts were visualized using bar and line charts generated with Origin 2024. Statistical analyses, including linear regression and Spearman correlation, were conducted using Prism 10. A *p*-value of < 0.05 was considered statistically significant, < 0.01 as highly significant, and < 0.001 as extremely significant. International collaboration networks were mapped using Scimago Graphica.

VOSviewer and CiteSpace served as the primary analytical tools [18] to perform co-occurrence analysis, keyword clustering, journal dual-map overlay, timeline visualization, and citation burst detection. In the generated network visualizations, nodes represent elements such as countries, institutions, authors, journals, or keywords. Connecting lines indicate collaborative or co-occurrence relationships, with line thickness proportional to the strength of association. A purple outer ring (in CiteSpace visualizations) denotes a centrality index; a centrality value exceeding 0.1 indicates that the node occupies a core and influential position within the network.

Additional journal metrics, including the 2022 Impact Factor (IF) and Journal Citation Reports (JCR) quartile, were sourced from the Journal Citation Reports. For CiteSpace analyses, the following parameters were applied: the log-likelihood ratio (LLR) algorithm for clustering, a *k*-value of 25 to specify the number of terms in each cluster, a time span from January 2007 to August 2025, and a time slice of one year.

## Results

### Publication trend analysis

Through a systematically designed search strategy and a rigorous screening process, 484 articles focusing on pain within the context of immune checkpoint inhibitor (ICI)–related adverse events were identified from the Web of Science Core Collection (WoSCC). A comprehensive analysis of these publications was conducted, and their disciplinary distribution was examined to elucidate the research landscape.

Analysis of the WoSCC subject categories assigned to these 484 articles reveals a distinct interdisciplinary pattern (Supplementary Table S2, summarized in Table 1). Oncology emerged as the core discipline, accounting for 53% of publications, underscoring the central focus on pain as a consequence of ICI-based anti-tumor therapy. Immunology (14%) and Pharmacology (10%) ranked second and third, respectively, indicating a concerted effort from the basic sciences to elucidate mechanisms and explore toxicity management strategies grounded in pharmacodynamics and pharmacokinetics.

**Table 1:** Ranking of Web of Science categories for selected 484 articles (TOP 20)

Rank	Web of Science categories	Record count	% of 484
1	Oncology	259	53.512
2	Immunology	68	14.050
3	Pharmacology Pharmacy	49	10.124
4	Medicine General Internal	47	9.711
5	Medicine Research Experimental	29	5.992
6	Rheumatology	25	5.165
7	Gastroenterology Hepatology	16	3.306
8	Clinical Neurology	15	3.099
9	Dermatology	14	2.893
10	Health Care Sciences Services	13	2.686
11	Endocrinology Metabolism	11	2.273
12	Respiratory System	11	2.273
13	Urology Nephrology	11	2.273
14	Radiology Nuclear Medicine Medical Imaging	9	1.86
15	Cell Biology	8	1.653
16	Surgery	8	1.653
17	Biochemistry Molecular Biology	7	1.446
18	Cardiac Cardiovascular Systems	7	1.446
19	Health Policy Services	5	1.033
20	Hematology	5	1.033

Notably, multiple clinical specialties contributed significantly to the corpus: Rheumatology (5.2%) addressed arthritis and myalgia; *Gastroenterology & Hepatology* (3.3%) involved abdominal pain and colitis-related pain; *Clinical Neurology* (3.1%) focused on neuropathic pain; *Dermatology* (2.9%) on pain associated with rash and pruritus; and *Endocrinology & Metabolism* (2.3%) on pain stemming from endocrine gland inflammation. This distribution mirrors the complexity of ICI-induced pain and its characteristic involvement of multiple organ systems. Furthermore, the substantial proportions represented by *General & Internal Medicine* (9.7%) and *Medicine, Research & Experimental* (6.0%) highlight that research in this domain extends beyond specialist perspectives to emphasize comprehensive patient management, translational research, and holistic care strategies. Contributions from *Cell Biology* (1.6%) and *Biochemistry & Molecular Biology* (1.4%) suggest a growing exploration into the fundamental cellular and molecular mechanisms underlying ICI-associated pain.

The extremely broad disciplinary coverage—spanning nearly 50 distinct WoS categories—highlights both the complexity of pain as an ICI-related adverse event and the critical importance of multidisciplinary collaboration (MDT) for its effective investigation and management. Current research has established a foundational understanding covering major affected organ systems and general management frameworks. Future investigations, while building upon these strengths, should seek deeper integration with disciplines such as anesthesiology & pain medicine, rehabilitation medicine, nursing, and psychiatry. Furthermore, leveraging emerging technologies to precisely characterize pain phenotypes and optimize personalized management strategies represents a vital direction for advancing the field.

### Publication volume and citation count

**Fig. 2:**
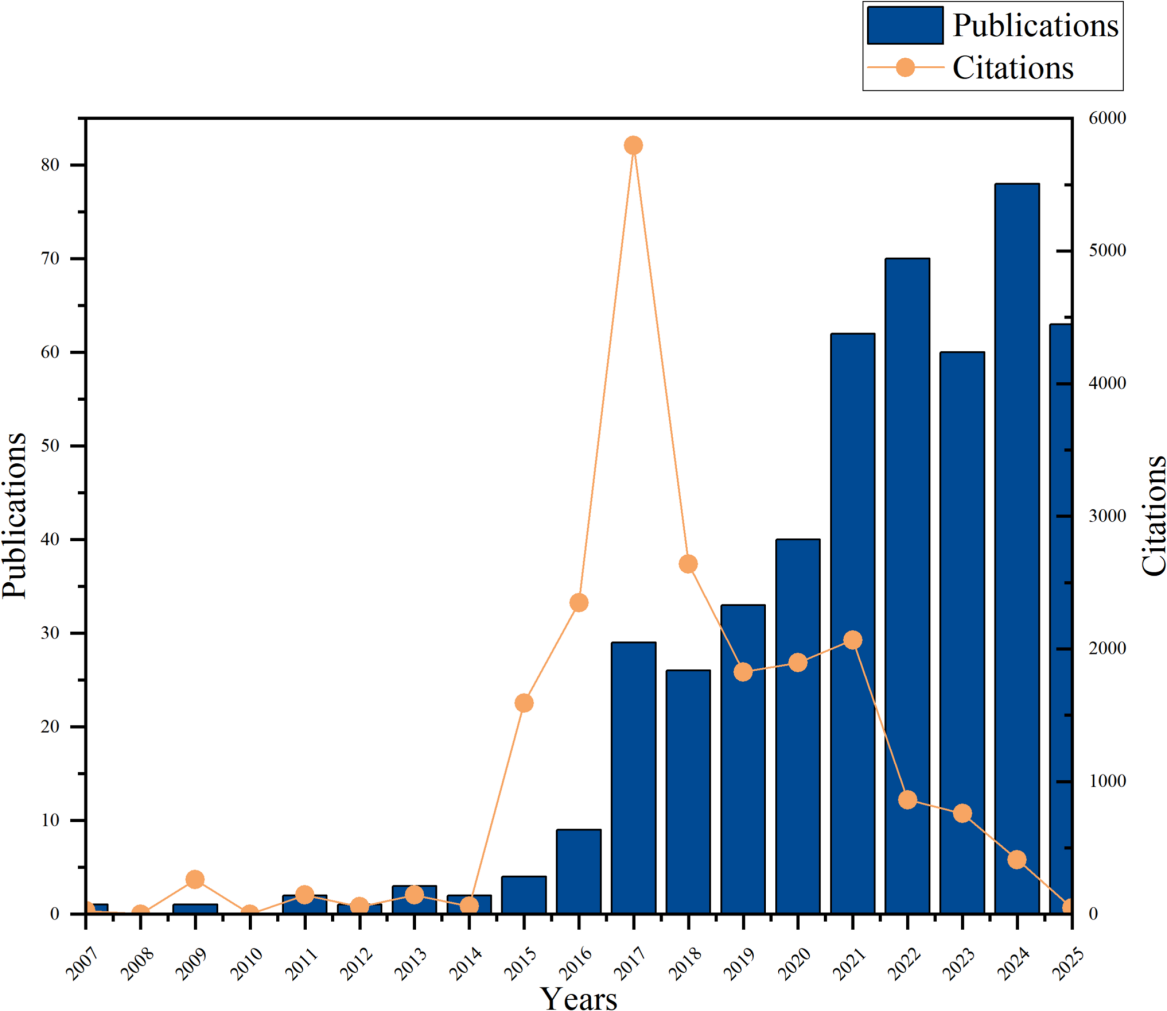
Annual publication output and cumulative citation trends for studies on pain spectrum in immune checkpoint inhibitor-related adverse events. Bar graph showing the number of publications per year. Line graph depicting the total citations received by publications from each year

The 484 included studies spanned publication years from 2007 to 2025. To visualize the temporal distribution of research activity and impact, the annual publication volume and the corresponding total citation counts were plotted (Fig. 2). Analysis of the chart reveals a clear, year-over-year increasing trend in the number of publications over time. This upward trend was statistically confirmed by linear regression analysis (*p* < 0.01). The derived prediction function for annual publications was Y = 4.621*X – 9291, with a correlation coefficient of *R*^2^ = 0.8458.

The year 2024 marked the peak in annual output with 78 publications. In contrast, the total citation count reached its highest point for papers published in 2017 (5794 citations), while citations for 2024 publications were comparatively lower (411 citations) at the time of analysis. Spearman correlation analysis indicated a statistically significant, moderate positive correlation between the publication year and the number of citations (*R* = 0.5037, 95% CI [0.04977, 0.7852], *p* = 0.03).

These patterns are closely aligned with the expanding clinical application of ICIs and the growing recognition of irAEs. The peak in citations for earlier publications (e.g., 2017) reflects the foundational impact of seminal studies, while the lower citation count for recent publications (e.g., 2024) is consistent with the known time-lag inherent in citation accumulation. Furthermore, it is important to note that data retrieval concluded in August 2025; therefore, the publication and citation counts for 2024 and 2025 are incomplete and likely underestimate the final yearly totals. Collectively, these data provide a quantitative basis for understanding the growth trajectory and evolving scholarly impact of this research field.

### Countries/region analysis

Researchers from 49 countries have contributed to the academic literature on pain-related adverse reactions to immune checkpoint inhibitors (ICIs). Supplementary Table S3 lists these countries by publication count and centrality, with the top 20 presented in Table 2.

**Table 2:** Global publication counts and centrality rankings of countries (top 20)

	Sort by article counts					Sort by centrality		
Rank	Country	Articles	Citations	TLS	Centrality	Country	Centrality	Articles
1	USA	184	15,175	232	0.35	USA	0.35	184
2	China	123	3568	54	0.00	UK	0.21	31
3	Japan	58	2508	67	0.02	Russia	0.13	7
4	France	56	6343	120	0.10	Spain	0.11	29
5	Germany	47	4448	146	0.02	France	0.10	56
6	Italy	38	4493	115	0.03	South Korea	0.08	15
7	United Kingdom	31	3130	115	0.21	Mexico	0.07	2
8	Spain	29	1824	123	0.11	Canada	0.07	26
9	Canada	26	3638	111	0.07	Australia	0.04	21
10	Australia	21	4309	103	0.04	Italy	0.03	38
11	Netherlands	19	3101	71	0.01	Switzerland	0.02	15
12	Belgium	15	1827	56	0.01	Germany	0.02	47
13	South Korea	15	1407	46	0.08	Japan	0.02	58
14	Switzerland	15	1559	38	0.02	South Africa	0.01	2
15	Greece	10	647	29	0.01	Israel	0.01	8
16	Turkey	9	91	11	0.00	Poland	0.01	5
17	Israel	8	3438	38	0.01	Greece	0.01	10
18	Brazil	7	921	15	0.00	Belgium	0.01	15
19	Russia	7	876	48	0.13	Netherlands	0.01	19
20	Poland	5	750	32	0.01	China	0.00	123

**Fig. 3:**
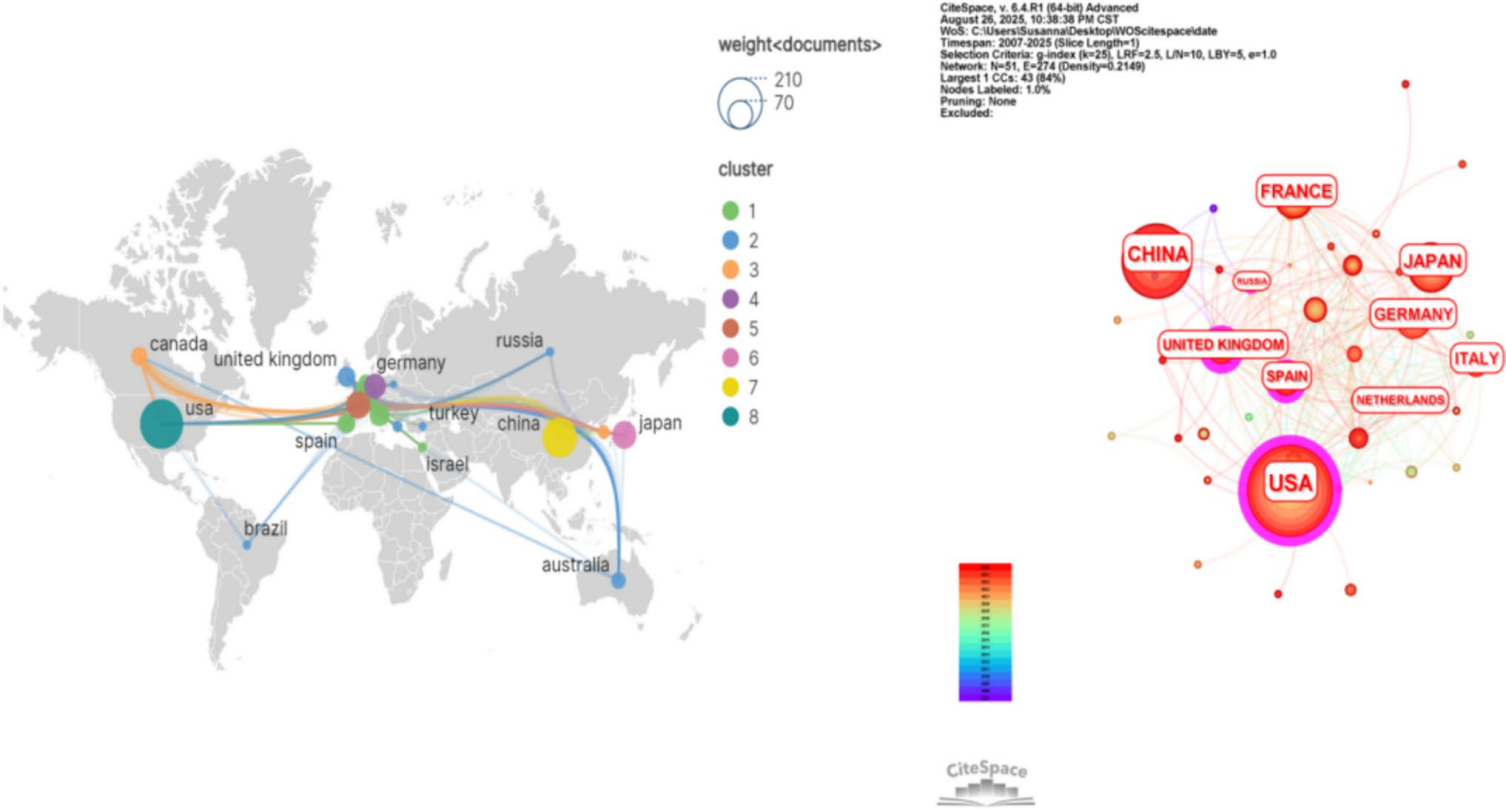
Geographical distribution and collaboration network of contributing countries. **A** Choropleth map illustrating the volume of publications by country. **B** Network visualization of international collaborations, with node size representing publication count and edge thickness reflecting collaboration strength

To visualize international collaboration, a national cooperation network was constructed using VOSviewer and Scimago Graphica (Fig. 3A). In this network, node size corresponds to the number of publications from each country, and connecting line thickness reflects the intensity of collaborative ties. The United States emerged with the highest publication output (184 articles, 38.0%) and the strongest collaborative links with other nations, underscoring its leading role in the field. Following the U.S., the top publishing countries were China (123 articles, 25.4%), Japan (58 articles, 11.9%), France (56 articles, 11.5%), and Germany (47 articles, 9.7%).

The structural role of countries within the global collaboration network was further assessed using CiteSpace to calculate centrality metrics (Fig. 3B). Nodes surrounded by a purple ring indicate a centrality value exceeding 0.1, with ring thickness proportional to centrality strength. This metric highlights countries that serve as crucial hubs in the research network. The USA exhibited the highest centrality, consistent with its leadership in both output and collaboration. The United Kingdom, despite a moderate publication count (31 articles), ranked second in centrality, indicating its important connective role. In contrast, while China ranked second in total publications, its relatively lower centrality value suggests there remains significant potential to strengthen its international collaborative engagement.

### Institutions analysis

**Fig. 4:**
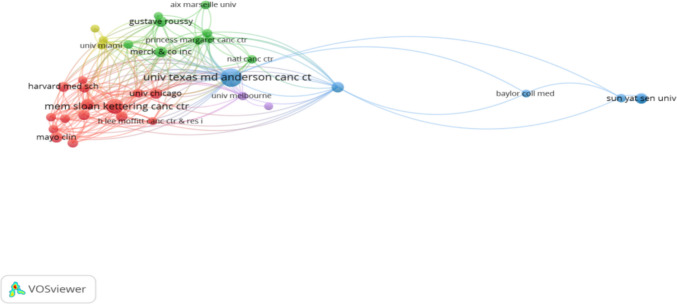
Network of institutional collaborations in pain-related ICI research. Nodes represent research institutions, sized by publication volume. Lines indicate collaborative ties, with thicker lines denoting stronger partnerships. The network highlights key hubs such as the University of Texas MD Anderson Cancer Center

The collaborative relationships among research institutions were mapped and analyzed using network visualization (Fig. 4). In the generated network, each node represents an institution, with its size proportional to the number of publications. Connecting lines indicate collaborative ties between institutions. The full network comprises 1220 distinct institutions; for visualization clarity, a refined network displaying 35 major nodes, 220 collaboration links, and 5 thematic clusters is presented. The University of Texas MD Anderson Cancer Center is prominently positioned as a representative core institution within this network.

The top 10 institutions by publication volume are listed in Table 3. A closer examination reveals that the majority of these high-output institutions are affiliated with the United States, including prominent universities, comprehensive cancer centers, and dedicated research institutes. This concentration indicates that U.S.-based academic entities have played a disproportionately significant role in shaping the published literature within this field. The data not only underscore the scientific influence of these leading institutions but also illustrate their pivotal function within global research collaboration. The outputs generated through these collaborative networks have contributed substantially to the advancement of the field and offer a valuable foundation for future investigations.

**Table 3:** Ranking of the top 10 institiutions by number of publications

Rank	Organization	Articles	Citations	TLS	Centrality	Country
1	University of Texas MD Anderson Cancer Center	34	2250	249	0.03	USA
2	Memorial Sloan-Kettering Cancer Center	23	3245	203	0.07	USA
3	Merck & Co., Inc	12	3405	141	0.06	USA
4	University of Washington	12	2949	107	0.04	USA
5	University of Chicago	11	3608	94	0.05	USA
6	Gustave Roussy Institute	11	2529	132	0.06	France
7	Mayo clinic	11	1452	74	0	USA
8	The Ohio State University	11	811	98	0.01	USA
9	Sun Yat-sen University	11	522	22	0	China
10	Dana-Farber Cancer Institute	10	1162	115	0.08	USA

### Authors analysis

A total of 3,876 authors were identified as direct contributors to the 484 publications analyzed. Among them, the six most productive authors, each having published five or more papers, are listed in Table 4. The leading contributor was Wang Yinghong with seven publications, followed by Zhang Haochi, Shatila Malek, Wang Chunjiang, Kostine Marie, and Johnson Donglasb, each with five publications.

**Table 4:** The 6 most productive authors

Rank	Author	Articles	Citations	TLS	Country
1	Wang Yinghong	7	59	13	USA
2	Zhang Haochi	5	33	12	USA
3	Shatila Malek	5	16	11	USA
4	Wang Chunjiang	5	20	4	China
5	Kostine Marie	5	49	0	France
6	Johnson Donglasb	5	633	1	USA

**Fig. 5:**
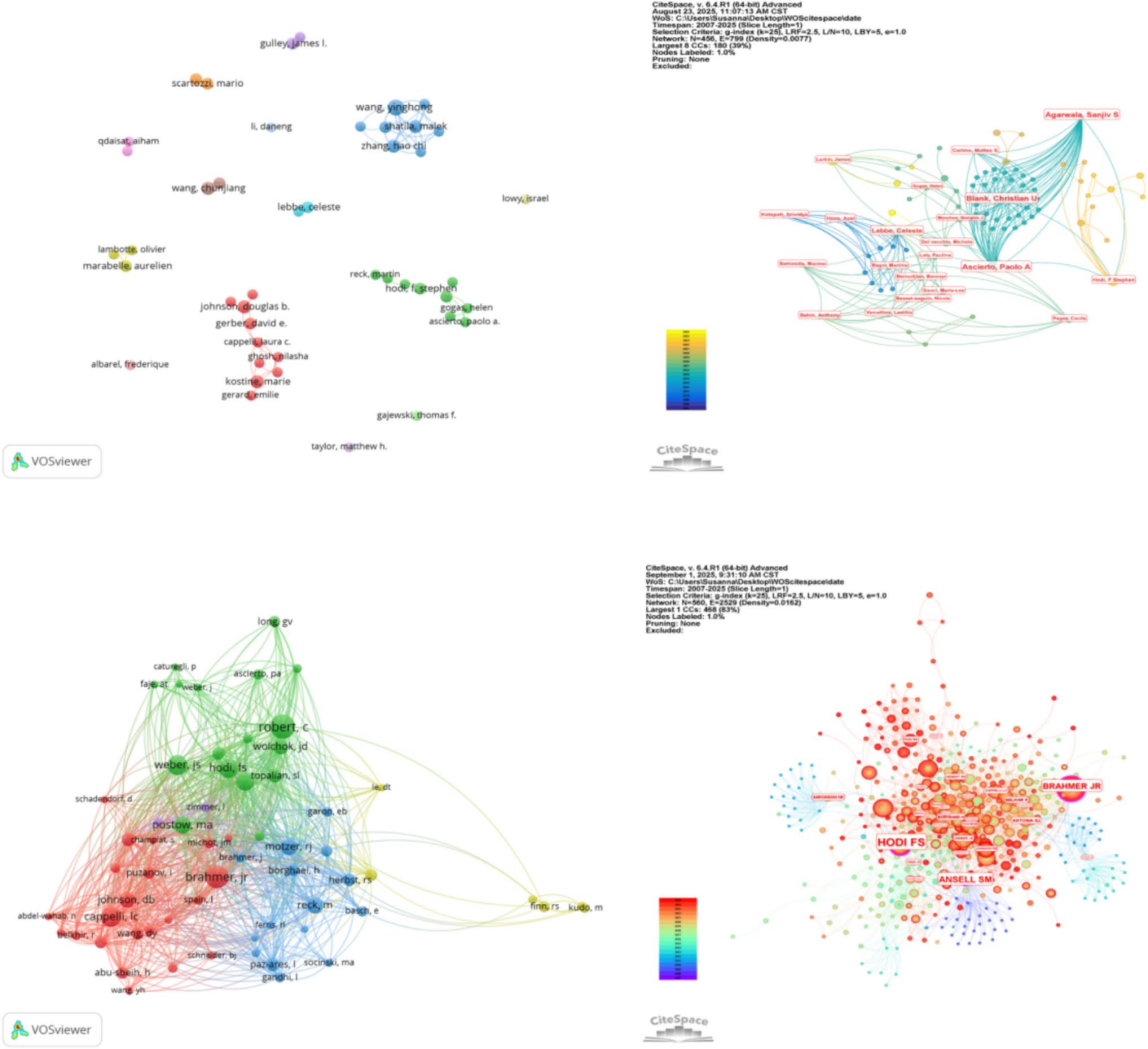
Author collaboration and co-citation networks. **A** Co-authorship network among prolific authors (≥ 3 publications). **B** Centrality analysis of the direct collaboration network. **C** Author co-citation network, reflecting shared intellectual foundations. **D** Centrality mapping within the co-citation network, identifying pivotal contributors to the field’s discourse

The collaborative relationships among the most active authors were mapped using VOSviewer. Figure 5A displays the co-authorship network of 45 authors who have each published at least three papers. The network resolves into three primary clusters: a red cluster (left) predominantly composed of researchers based in the USA, a green cluster (right) mainly comprising researchers from Germany, and a blue cluster (upper section) also largely consisting of U.S.-based researchers. The analysis indicates strong collaborative ties within each cluster, while connections between clusters—representing international collaborations—remain relatively sparse.

Further analysis using CiteSpace (Fig. 5B) assessed the centrality of authors within the collaboration network. The centrality values for all authors were found to be below the threshold of 0.1, indicating that no single author currently serves as a dominant connective hub in the direct collaboration network.

In contrast, author co-citation analysis, which maps the frequency with which two authors are cited together by other papers, reveals a different and more integrated intellectual structure. As shown in Figs. 5C and D, the most co-cited authors were Robert C, Brahmer Jr, and Weber JS, with co-citation frequencies of 135, 120, and 98, respectively. This network exhibits a high degree of connectivity, suggesting a mature and shared knowledge base. Notably, within this co-citation network, Hodi FS (centrality 0.19), Ansell SM (centrality 0.15), and Brahmer Jr (centrality 0.14) emerged as pivotal nodes, each with a centrality value exceeding 0.1. This highlights their foundational influence on the field’s discourse, despite their more moderate centrality in the direct authorship network. The well-established and tightly knit structure of the co-citation network underscores the existence of deep conceptual synergies and a common scholarly foundation among researchers in this domain.

### Analysis of top journals

The 484 publications were disseminated across 211 distinct academic journals. Table 5 lists the top 10 most prolific journals, detailing their publication volume, country of origin, 2022 Impact Factor (IF), Journal Citation Reports (JCR) quartile, total citation count, and total link strength (TLS). The publishing bodies of these leading journals are predominantly located in Switzerland and the USA. *Frontiers in Oncology* was the most prolific journal with 23 articles, followed by *Frontiers in Immunology* (20 articles) and *Lancet Oncology* (18 articles). Among the top 10, eight journals (*Frontiers in Oncology*, *Lancet Oncology*, *Journal for Immunotherapy of Cancer*, *Journal of Oncology Pharmacy Practice*, *Clinical Cancer Research*, *Cancers*, *Melanoma Research*, and *The Oncologist*) primarily focus on oncology, while two (*Frontiers in Immunology* and *Journal for Immunotherapy of Cancer*) emphasize immunology. The journal *Medicine* represents the pharmacy discipline. Most of these top journals are ranked in JCR Q1 or Q2 categories, reflecting their high academic impact and recognition.

**Table 5:** Top 10 relevant journals

Rank	Journal title	Articles	Country	IF	JCR	Total citations	TLS
1	Frontiers in Oncology	23	Switzerland	3.3	Q2	352	20
2	Frontiers in Immunology	20	Switzerland	5.9	Q1	101	11
3	Lancet Oncology	18	England	35.9	Q1	5887	17
4	Journal for Immunotherapy of Cancer	16	USA	10.6	Q1	793	8
5	Journal of Oncology Pharmacy Practice	14	USA	5.9	Q1	70	5
6	Clinical Cancer Research	11	USA	10.2	Q1	969	3
7	Cancers	9	Switzerland	4.4	Q2	129	8
8	Medicine	9	USA	1.4	Q2	35	2
9	Melanoma Research	9	USA	2.1	Q3	79	5
10	Oncologist	9	USA	5.8	Q2	396	9

**Fig. 6:**
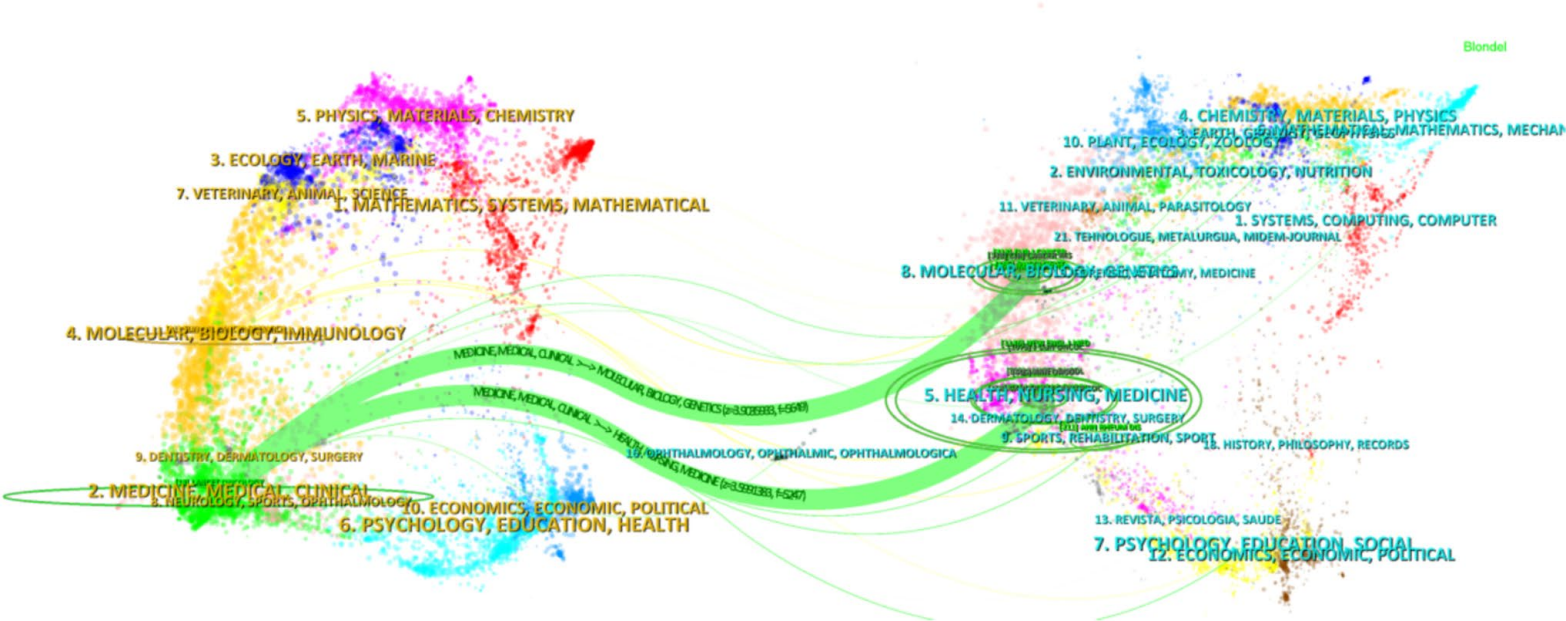
Dual-map overlay of journals illustrating interdisciplinary citation flow. Citing journals are primarily clustered in clinical and medical disciplines, while cited journals are concentrated in molecular biology and genetics, underscoring the translational nature of pain research in ICI-related adverse events

A dual-map overlay of journals, generated using CiteSpace (Fig. 6), illustrates the interdisciplinary citation flow within this research domain. The analysis reveals two primary citation pathways. Publishing journals are largely concentrated in fields related to medicine, medical technology, and clinical practice. In contrast, the cited references predominantly originate from the domains of molecular biology and genetics. This distinct cross-disciplinary citation pattern underscores the breadth and depth of research on the pain spectrum in ICI-related adverse events. It highlights the field’s inherently multidisciplinary nature, bridging fundamental mechanistic discovery at the molecular level with the practical challenges of recognition and management in clinical oncology.

### Cited references and co-cited references analysis

Within the corpus of 484 articles on pain in immune checkpoint inhibitor (ICI)–related adverse events, 43 publications have been cited more than 100 times. According to the data presented in Table 6, the most cited article is the 2016 study by R. Nanda, L.Q.M. Chow et al., published in the *Journal of Clinical Oncology*, with a total of 1,624 citations. This was followed by the 2015 study by A. Ribas, I. Puzanov, R. Dummer et al. in *Lancet Oncology*, with 1,281 citations.

**Table 6:** Top 10 cited articles

Rank	First author	Journal	Times cited	Year	DOI
1	R. Nanda	Journal of Clinical Oncology	1624	2016	10.1200/jco.2015.64.8931
2	A. Ribas	Lancet Oncology	1281	2015	10.1016/s1470-2045(15)00083-2
3	L. Khoja	Annals of Oncology	679	2017	10.1093/annonc/mdx286
4	M. A. Davies	Lancet Oncology	565	2017	10.1016/s1470-2045(17)30,429-1
5	S. P. D’Angelo	Lancet Oncology	552	2018	10.1016/s1470-2045(18)30,006-8
6	N. van Zandwijk	Lancet Oncology	541	2017	10.1016/s1470-2045(17)30,621-6
7	J. R. Brahmer	Journal for Immunotherapy of Cancer	532	2021	10.1136/jitc-2021-002435
8	R. Gutzmer	Lancet	497	2020	10.1016/S0140-6736(20)30,934-X
9	S. Baxi	Bmj-British Medical Journal	458	2018	10.1136/bmj.k793
10	S. Gettinger	Journal of Clinical Oncology	427	2016	10.1200/jco.2016.66.9929

**Fig. 7:**
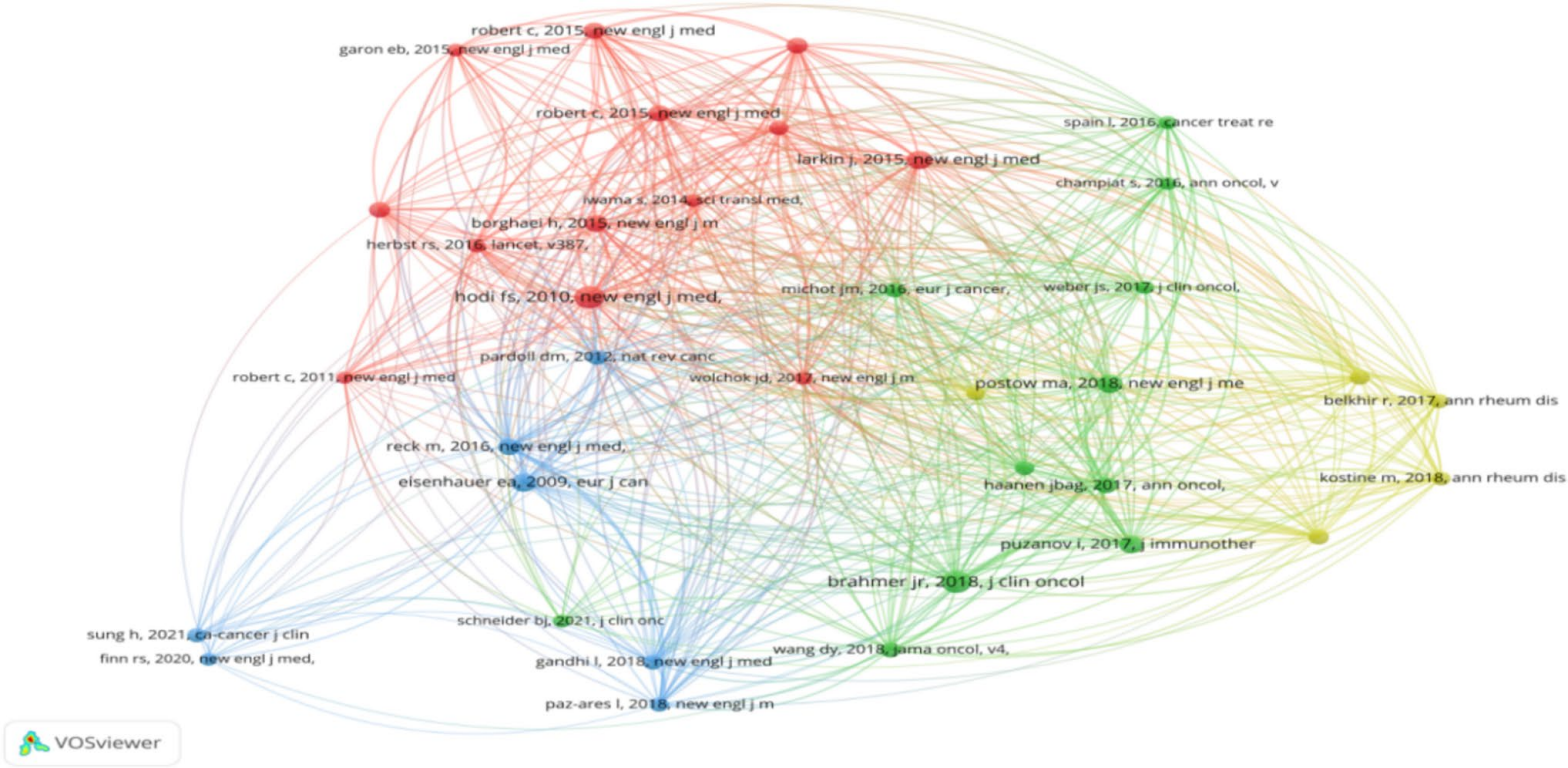
Citation dynamics and thematic clusters of co-cited references. **A** Top 25 references exhibiting the strongest citation bursts, highlighting periods of high influence. **B** Co-citation network clustered into four major themes: direct ICI-induced pain, pathogenesis and prediction, interaction with tumor/conventional therapy pain, and pain in patients with pre-existing autoimmune conditions

The growth trajectory of citations for the top 25 most cited papers in this field is illustrated in Fig. 7A. The significant and sustained increase in their citation volume reflects the emergence and evolution of research hotspots. Citation activity began to rise notably around 2010. The period between 2012 and 2021 was characterized by frequent shifts in citation hotspots, indicating rapid and dynamic development. Since 2021, a new cycle of research foci has continued to emerge and remains active, demonstrating that the study of pain within ICI-related adverse events maintains consistent and high academic interest.

A co-citation analysis, visualized using VOSviewer (Fig. 7B), identified four major thematic clusters, delineating the core intellectual structure of the field:

1. The Red Cluster focuses on pain complications directly induced by cancer immunotherapy.
2. The Green Cluster centers on immunotherapy-related cytokine release syndrome (CRS)—which can cause systemic inflammation and pain—and includes research on biomarkers for predicting patient susceptibility to immune-related pain.
3. The Blue Cluster investigates pain related to the primary tumor (e.g., bone metastases, nerve compression) and pain caused by conventional treatments like chemotherapy and radiotherapy, exploring their interaction with immunotherapy.
4. The Yellow Cluster is directly concerned with pain induced after immunotherapy in cancer patients with pre-existing autoimmune or inflammatory diseases.

These clusters collectively map the current research landscape, highlighting key directions such as the characterization of pain subtypes, investigation of pathogenesis and risk prediction, understanding interactions between different pain etiologies, and managing pain in vulnerable patient subgroups.

### Research hotspot keywords analysis

**Fig. 8:**
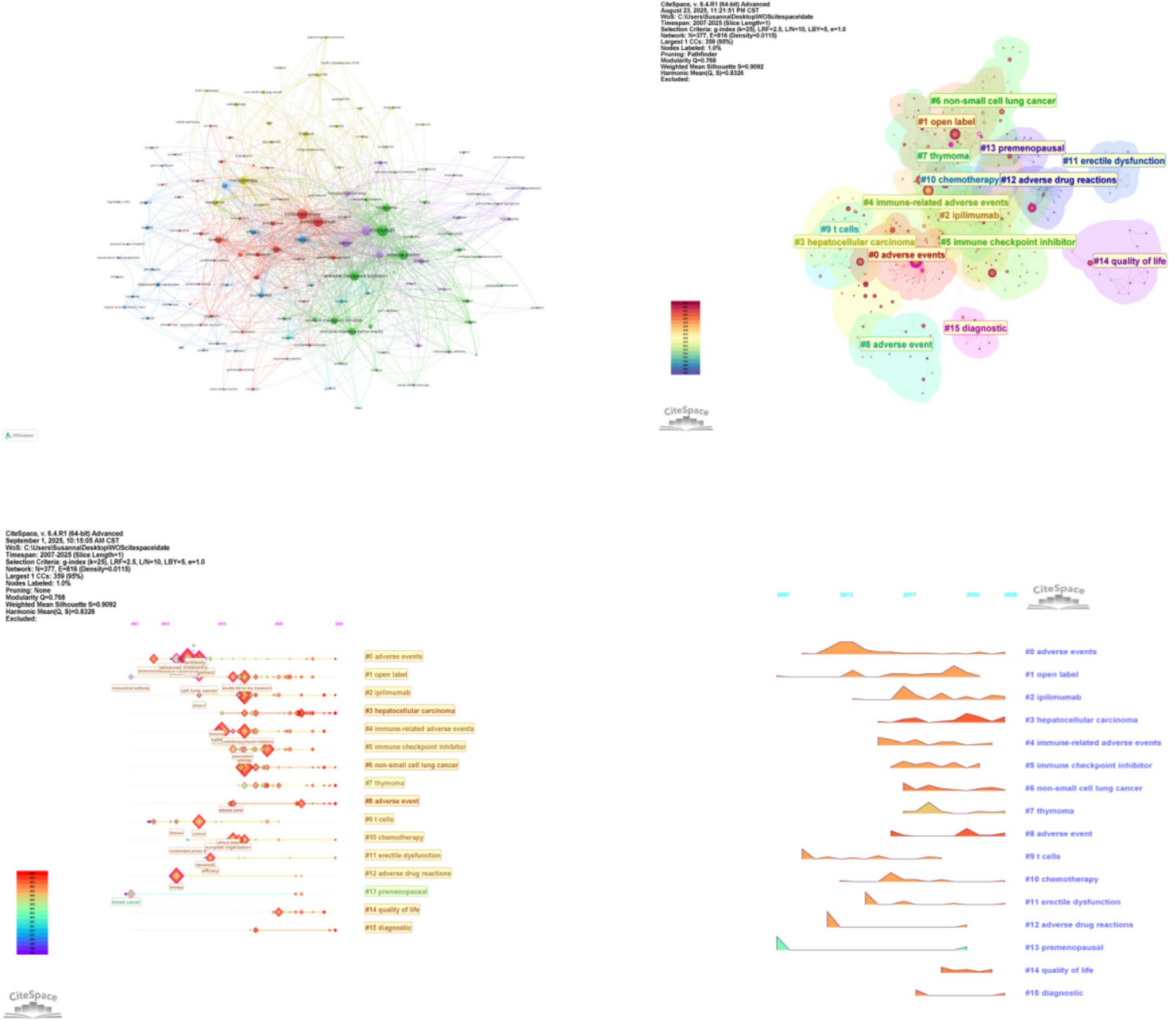
Keyword co-occurrence, clustering, and evolution over time. **A** Network of high-frequency keywords, with node size indicating frequency and links representing co-occurrence. **B** Thematic clustering of keywords, revealing core research directions. **C** Timeline view of keyword clusters, showing the emergence and persistence of research themes. **D** Landscape view highlighting the temporal prominence of key topics such as hepatocellular carcinoma and quality of life

Keyword analysis serves to uncover the intrinsic connections between research topics and to delineate evolving trends within a specific field. From the 1744 keywords identified in this study, 29 occurred more than 20 times. The keyword co-occurrence network, constructed using VOSviewer (Fig. 8A), visualizes these relationships. In this map, node size corresponds to a keyword’s frequency of occurrence, and the thickness of connecting lines reflects the strength of association between terms. Among all keywords, “nivolumab” appeared most frequently, followed by “immunotherapy,” “pembrolizumab,” “ipilimumab,” and “adverse events.” When search-specific terms are excluded, the top five keywords by co-occurrence frequency, in descending order, are “cancer,” “melanoma,” “chemotherapy,” “management,” and “safety.”

To identify thematic research structures, a keyword clustering analysis was performed using CiteSpace (Fig. 8B). The validity of the clustering is supported by a modularity (Q) value of 0.768—significantly above the 0.4 threshold—indicating a robust cluster structure. An average silhouette (S) value of 0.9092, exceeding 0.5, confirms excellent cluster homogeneity and convincing results. The analysis yielded 15 distinct clusters. After excluding generic terms (e.g., “open label”) and the original search keywords, the remaining clusters reveal the field’s principal research directions, which include: specific cancer types (hepatocellular carcinoma, non-small cell lung cancer, thymoma), mechanistic investigations (“T cells”), treatment modalities (“chemotherapy”), specific dysfunctions (“erectile dysfunction”), patient subgroups (“premenopausal”), and core outcomes (“quality of life,” “diagnostic”). This indicates that research primarily focuses on disease-specific presentations, underlying mechanisms, diagnostic approaches, and impacts on patient well-being.

To trace the temporal evolution of research foci, a keyword timeline view (Fig. 8C) and a keyword time peak map (Fig. 8D) were generated. Figure 8C displays the 15 clusters along a timeline, where node size indicates keyword frequency within the cluster and colored lines represent co-occurrence links between clusters. Clusters #1, #9, and #13 formed earlier and have sustained attention, while clusters centered on “adverse events” and “diagnosis” represent more recent hotspots. Figure 8D illustrates that “hepatocellular carcinoma” maintained consistent research interest from its emergence around 2016 until approximately 2022. Clusters such as “lung cancer,” “hepatocellular carcinoma,” and “quality of life” have demonstrated persistent popularity, with interest in “hepatocellular carcinoma” seeing a notable increase in recent years.

## Discussion

Immune checkpoint inhibitors (ICIs) represent a transformative class of cancer immunotherapeutics that reinvigorate antitumor T-cell activity by blocking inhibitory signals within the tumor microenvironment [19, 20]. Since the initial approval of the anti-CTLA-4 antibody ipilimumab for advanced melanoma in 2011 [21], the indications for ICIs have expanded across numerous advanced malignancies, substantially improving overall survival in these populations [22, 23]. Nevertheless, the management of irAEs remains critical to achieving optimal therapeutic outcomes. Among irAEs, pain constitutes a particularly frequent, complex, and quality-of-life-limiting manifestation that poses distinct clinical challenges [24]. ICI-related pain is not a singular symptom; rather, it presents as a complex multidimensional spectrum due to its varied anatomic sites, diverse phenotypes, multifactorial mechanisms, fluctuating temporal course, and considerable diagnostic complexity [25]. Against this background, we performed a comprehensive bibliometric analysis to visually delineate the research landscape, identify current focal points, and forecast emerging directions in this field.

By reviewing literature related to the pain spectrum in ICI-associated adverse events, we identified 484 relevant articles published from January 2007 to August 2025, and analyzed research trends based on annual publication volume and citation counts. From 2007 to 2015, research on ICI-related pain was in its nascent stages, characterized by low annual publication and citation rates. This reflects the initial clinical focus on the oncological efficacy of ICIs, with limited recognition of irAEs, particularly pain. Beginning in 2016, this domain exhibited exponential growth, with a marked increase in publications through 2023, culminating in a peak in 2024. This surge in research intensity is largely attributable to two key factors. First, the remarkable efficacy of PD-1/PD-L1 inhibitors in treating major cancers, such as non-small cell lung cancer, melanoma, and renal cell carcinoma led to their rapid and widespread clinical adoption. Consequently, irAEs, acting as a “double-edged sword” became an unavoidable clinical challenge, directly stimulating scientific inquiry into issues like ICI-related pain. Second, the successive publication of irAE management guidelines by organizations including ASCO and ESMO provided clinicians with standardized protocols and crucially, systematically outlined the limitations of current knowledge and identified blank areas requiring future research. Thus, both “clinical demand” and “academic guidance” jointly catalyzed this research surge. The apparent decrease in publication volume after 2024 likely does not indicate a decline in the field’s relevance. It is essential to note that data retrieval concluded in August 2025, resulting in incomplete data for that year and a potential underestimation of the final count. Citation counts peaked around 2017–2018, likely reflecting the profound impact of seminal early studies on subsequent research. This pattern is closely linked to the broadening application of ICIs across oncology and the increasing clinical prominence of irAEs. Finally, it is important to recognize that citation accumulation exhibits a clear time-lag effect; the lower citation counts for literature published in 2024 are therefore normal and should not be directly compared with those of earlier publications.

Geographically, the USA led in publication output (38.0%), centrality, and international collaborative linkages, followed by China, Japan, France, and Germany. Eight of the top 10 most productive institutions were U.S.-based, underscoring the country’s dominant role. The collaborative model between the University of Texas MD Anderson Cancer Center (U.S.) and Gustave Roussy Institute (France) exemplifies how combining expertise in supportive care and immuno‑toxicity can advance the study of complex pain phenotypes [26, 27]. Although China ranked second in output, its relatively lower centrality suggests room for enhanced global collaboration.

Through the analysis of the author collaboration network, we found that there is a certain degree of collaboration among authors, but an extensive international collaboration network has not yet been formed. The potential reasons for the low density of cooperation density among authors may be constrained by various real-world factors. Policy obstacles including restrictions on data sharing and differences in ethical review processes hinder cross-border collaboration and integration of research data. Financial gaps also pose a significant challenge, as multinational collaborative research often requires greater financial support. However, disparities in research funding systems across countries/regions impede the efficient flow of resources. Language limitations similarly play a notable role; although most academic papers are published in English, language barriers among researchers during stages such as study design, data collection, and interpretation of results may reduce the efficiency of collaboration. Three distinct clusters are clearly visible in Fig. 5A, with authors within clusters collaborating closely, while inter-cluster collaboration remains relatively sparse. The centrality of all authors does not exceed 0.1, a phenomenon also reflected in the node distribution in Fig. 5A. Notably, most of the top 6 authors by publication volume are from the University of Texas MD Anderson Cancer Center (USA). Their research mainly focuses on exploring the importance of pain as an early identification indicator and investigating how to predict toxic reactions through Patient-Reported Outcomes to shorten the time window for clinical intervention [28], which has brought a new perspective to the field of pain spectrum in immune-related adverse events (irAEs).

In the author co-citation analysis, although Robert C’s centrality value did not reach 0.1, he still topped the list in citation count. Hodi Fs had the highest centrality value, reaching 0.19. His research focuses on pain associated with severe but uncommon neurological immune-related adverse events (irAEs) related to immune checkpoint inhibitors, proposing that clinicians should use established algorithms for timely diagnosis and management to minimize severe complications of these neurological irAEs [29].

According to the data in Table 5, Lancet Oncology (IF = 35.9, Q1), “Clinical Cancer Research” (IF = 10.2, Q1), and “Journal for Immunotherapy of Cancer” (IF = 10.6, Q1) ranked among the top three in terms of citation frequency in this field. With a total citation count of 5887, “Lancet Oncology” took the lead among the top ten journals in publication volume, highlighting its academic status in this field. The high influence and academic recognition of these journals provide an important publication platform for research in this field. Journal analysis offers researchers subsequent research directions and a basis for journal selection; scholars can determine research priorities based on the thematic directions of high-impact journals and select appropriate journals for submission.

As can be seen from Fig. 6, cited literature mostly appeared in the fields of molecular biology and genetics, while publishing institutions were mainly concentrated in disciplinary areas such as medicine, medical technology, and clinical practice. This interdisciplinary citation pattern reveals the breadth and depth of research on the pain spectrum in immune checkpoint inhibitor (ICI)–related adverse events, highlighting the multidisciplinary nature of this field and its core position in clinical research. It also confirms the translation of this field from the exploration of basic mechanisms at the molecular and genetic levels to addressing the challenges of pain recognition and management in clinical practice [2, 30].

In the analysis of literature co-citation relationships, which serves as a reliable indicator reflecting research foundations, the study found that, according to the data in Table 6, the most cited paper was the research by R. Nanda; L. Q. M. Chow et al., published in *the Journal of Clinical Oncology *(IF = 41.9, Q1) in 2016, with 1624 cumulative citations. This study was an early exploration of KEYNOTE-012 as a PD-1 inhibitor in the triple-negative breast cancer (TNBC) field. Although the recording of pain-related adverse events was limited by the Ib phase study design, some details hold special significance for the analysis of the “pain spectrum.” The study clarified that immune-related pain may be associated with treatment cycles, emphasizing the need to take the risk of chronic pain into consideration. It also proposed that “extensive pre-treatment” may be a potential confounding factor for immune-related pain, highlighting the importance of distinguishing residual toxicities from chemotherapy superimposed on immunotherapy. Additionally, it pointed out the insufficient attention to “mild pain affecting quality of life” in early immunotherapy and discussed the management approaches for severe headaches caused by immune-related neurological irAEs such as aseptic meningitis and encephalitis [31]. The study by A. Ribas, I. Puzanov, R. Dummer et al., published in the Lancet Oncology in 2015, ranked second with 1281 cumulative citations. This paper noted that immune-mediated adverse events (AEs) are managed with corticosteroids, which can alleviate inflammatory pain such as arthralgia and myalgia. However, long-term use may lead to muscle weakness and osteoporosis, thereby exacerbating pain. It proposed the need to balance the rapid control of irAEs with avoiding the negative impact of long-term steroid use on pain [32], identifying current clinical challenges and laying an important foundation for subsequent research.

Through visualization analysis of literature co-citation, it was found that current research in this field focuses on the following four core directions: (1) types of pain directly induced by cancer immunotherapy; (2) pathogenesis and risk prediction of pain in immune checkpoint inhibitor-related adverse reactions; (3) interaction between cancer or conventional treatment-related pain and pain induced by cancer immunotherapy; (4) pain risk in special populations with autoimmune diseases or underlying inflammatory conditions after cancer immunotherapy. These findings point out the direction for future refined research on pain subtypes, establishment of prediction models, and development of individualized management strategies.

Keywords are important indicators of research hotspots and frontiers. Visualization of high‑frequency keywords (Fig. 8A) shows that “nivolumab,” “immunotherapy,” “pembrolizumab,” “ipilimumab,” and “adverse events” occur most frequently. The prominence of “nivolumab,” “pembrolizumab,” and “ipilimumab” not only confirms their status as core ICI agents but also suggests their notable association with or high reporting frequency of pain adverse events in clinical practice, prompting researchers to pay particular attention to the pain spectrum characteristics and mechanistic differences among these commonly used ICIs. As core nodes, “immunotherapy” and “adverse events” further indicate that pain constitutes an integral and highly focused branch within overall ICI toxicity research. Excluding search terms, the top five keywords by co‑occurrence frequency are “cancer,” “melanoma,” “chemotherapy,” “management,” and “safety.” “Cancer” and “melanoma” reflect disease specificity, with melanoma being a pioneering field for ICI application [33]. Further exploration reveals that pain in melanoma primarily manifests as rheumatic and musculoskeletal irAEs, with a relatively high incidence (5–20%). Notably, when CTLA‑4 inhibitor (ipilimumab) is combined with a PD‑1 inhibitor, pain may occur early or late, and some cases progress to chronicity [34, 35]. In non‑small cell lung cancer (NSCLC), pain types are more complex and diverse, with neuropathic pain and bone pain being prominent [36]. In head and neck squamous cell carcinoma (HNSCC), local inflammation‑related pain is significant, especially when combined with radiotherapy, requiring differentiation from tumor invasion or post‑radiotherapy neuralgia [37]. Hepatocellular carcinoma, gastric cancer, pancreatic cancer, and renal cell carcinoma are characterized mainly by visceral pain and bone pain, with relatively fewer reports of rheumatic pain compared with other cancer types [38]. “Chemotherapy” indicates that current research strongly focuses on pain issues during combined or sequential ICI‑chemotherapy regimens. A study by West H. et al. comparing atezolizumab plus carboplatin/nab‑paclitaxel versus chemotherapy alone found higher incidence rates of arthralgia (20% vs 14%), myalgia (18% vs 12%), and peripheral neuropathy (48% vs 43%) in the combination group [39]. ESMO guidelines note that chemotherapeutic agents such as taxanes, platinum compounds, and vinca alkaloids are common causes of painful neurotoxicity (e.g., peripheral neuropathy), and their combination with ICIs may exacerbate neuropathy or complicate differential diagnosis [40]. This highlights that the superimposition of chemotherapy‑induced pain and ICI‑related adverse events constitutes a key difficulty and hotspot in clinical management. The high co‑occurrence of “management” and “safety” underscores the need to effectively identify, assess, prevent, and manage ICI‑induced pain while preserving ICI efficacy, thereby ensuring patient safety and treatment tolerance.

Figure 8B presents a keyword clustering map generated using CiteSpace. The high modularity (Q = 0.768) and silhouette (S = 0.9092) values confirm the reliability of the clustering and strongly support that pain‑related adverse reactions to immunotherapy constitute a research field with clear internal structure, distinct themes, and high focus. Research themes are not scattered but form an organically connected knowledge network. The presence of hepatocellular carcinoma, non‑small cell lung cancer, thymoma, etc., in the clustering map illustrates that research is expanding from melanoma to tumor types with broader ICI application, indicating researchers recognize pain as a cross‑tumor issue and reflecting increased research breadth. The independent cluster “T cells” strongly points to an active area of basic and translational research. Investigators are deeply exploring the core role of immune mechanisms—such as T‑cell overactivation, cytokine storms (e.g., IL‑6, TNF‑α), and autoimmune‑like reactions (e.g., arthritis, myositis)—in pain pathogenesis. Studies by Cappelli L.C. et al. [34] found massive infiltration of CD8+ T cells and macrophages in synovial tissue of patients with ICI‑induced arthritic pain, suggesting T‑cell‑mediated local inflammation as a direct cause of joint pain. Braaten T. J. et al. [41] confirmed that activated T cells (CD4+/CD8+) and plasma cells persist in patients’ joints even after treatment discontinuation, correlating with chronic pain. Elevated IL‑6 levels are significantly associated with ICI‑induced pain [42], laying a theoretical foundation for cytokine‑targeted interventions. Braun D.A. et al. reported that factors such as TNF‑α and IL‑6 contribute to neuropathic pain by activating glial cells and neuronal TRP channels [43]. Pathak R. et al. [44] detected muscle‑specific autoantibodies in ICI‑induced myositis, where T‑cell‑mediated myofiber necrosis directly causes pain. Aberrant T‑cell activation appears to be a common initiating event in ICI‑related pain, leading to multi‑organ pain syndromes through three core mechanisms: direct tissue infiltration, cytokine storm, and autoimmune attack. Targeted therapies against T cells and their downstream effectors (IL‑6, TNF‑α, IL‑17) have shown promise in clinical practice [45] including anti‑IL‑6R for rapid relief of IL‑6‑driven joint pain [46], anti‑TNF‑α for refractory arthritis and enteritis‑related pain [47], JAK inhibitors that block the JAK‑STAT pathway and downstream cytokine production in refractory rheumatic irAEs [48], and B‑cell depletion for severe neuromuscular pain with autoantibodies [49]—all aimed at identifying potential effective “steroid‑sparing strategies.” Clusters such as “premenopausal” and “erectile dysfunction” highlight attention to specific populations, pointing to pain resulting from ICI effects on the endocrine system (e.g., hypophysitis, ovarian dysfunction, orchitis) or the potential influence of hormonal status on pain perception and ICI responsiveness. The “quality of life” cluster underscores the severe impact of pain on patients’ well‑being, while “diagnostic” reflects that accurate identification and differentiation of ICI‑related pain remains a key challenge, encompassing early identification markers (clinical symptoms, imaging, laboratory markers) and the definition/classification of complex pain syndromes. Diagnostic difficulties directly affect subsequent management decisions.

For future research directions, current research has confirmed the core roles of T-cell overactivation and cytokine storms in the occurrence of pain, but issues such as the specific pathways of neuro-immune interaction and the specific immune cell subsets behind different pain phenotypes (such as neurogenic and inflammatory) still require systematic analysis. In future, it may be possible to combine single-cell sequencing, spatial transcriptomics, and other technologies to draw a dynamic map of the pain-related immune microenvironment to further deepen research in this field.

Examination of the keyword timeline (Fig. 8C), time–peak map (Fig. 8D), and burst detection analysis highlights a notable surge in research interest in hepatocellular carcinoma. Patients with liver cancer often have underlying cirrhosis, and hepatic dysfunction may influence drug metabolism and toxicity. Studies report unique pain syndromes following immunotherapy in liver cancer [50]. Treatment‑related hepatitis pain must be differentiated from abdominal pain associated with portal vein tumor thrombosis (PVTT). For instance, immune‑mediated hepatitis tends to present as persistent right‑upper‑quadrant dull pain with elevated liver enzymes, whereas pain from tumor thrombosis often worsens with positional changes and is accompanied by signs of portal hypertension. Imaging showing arterial enhancement of PVTT suggests tumor thrombosis [51], while elevated IgG4 supports a diagnosis of immune‑mediated hepatitis.

**Fig. 9:**
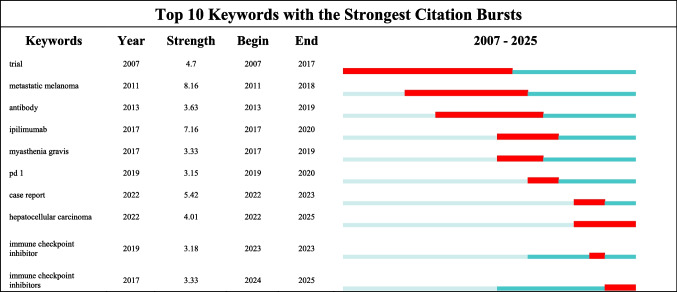
Top 10 keywords with the strongest citation bursts, indicating shifting research hotspots over time

Keyword burst analysis (Fig. 9) demonstrates that research hotspots in ICI‑related pain exhibit clear temporal evolution. The keyword with the highest burst strength, “metastatic melanoma,” reflects early research emphasis on ICI adverse events in melanoma treatment. In recent years, the emergence of “case report” and “hepatocellular carcinoma” indicates growing attention to case‑series accumulation and pain‑related adverse events in newer indications such as hepatocellular carcinoma. Sustained research on “pd 1” and “immune checkpoint inhibitors” reveals enduring focus on pain management in PD‑1 inhibitors and ICIs overall, guiding exploration of pain mechanisms and intervention strategies.

## Limitations and shortcomings

Several limitations of this bibliometric study should be acknowledged. First, the reliance solely on the Web of Science Core Collection (WoSCC) introduces a potential source of systematic bias. While WoSCC is authoritative and widely used for bibliometric analysis, it may not capture all relevant literature, particularly clinical pain studies indexed in other specialized databases, regional reports, or non‑English publications. Variations in terminology and indexing practices across databases further limit the generalizability of our findings; thus, the conclusions primarily reflect academic trends within the WoSCC ecosystem.

Second, the fragmentation and inconsistency of pain-related terminology in the literature likely lead to an underestimation of the true incidence and diversity of ICI-related pain phenotypes. This heterogeneity complicates systematic retrieval and synthesis. Third, the scarcity of long-term follow-up data (only 2.4% of studies reported follow-up ≥ 5 years) restricts robust analysis of chronic pain trajectories, late-onset syndromes, and long-term outcomes after ICI therapy.

Finally, bibliometric analysis is inherently descriptive and reflects research activity at a specific point in time. It identifies trends and intellectual structures but cannot ascertain causal relationships or clinical efficacy. The dynamic nature of the field means that emerging topics may not be fully captured in our dataset, which was frozen in August 2025.

To address these limitations, future work should aim to (1) develop a standardized, consensus‑based lexicon for ICI‑related pain to improve literature retrieval and cross‑study comparability; (2) expand searches to include multilingual and regional specialty databases; (3) promote prospective registries and real‑world evidence initiatives to collect longitudinal pain‑specific data; and (4) integrate bibliometric insights with systematic reviews and meta‑analyses to provide a more comprehensive evidence base.

## Conclusion

This bibliometric analysis outlines the evolving landscape of ICI‑related pain research. Pain constitutes a core toxicity spectrum that critically affects treatment tolerance and quality of life. While the USA and China lead in output, broader international collaboration is needed to address regional evidence gaps. Moving forward, priority should be placed on deepening global data sharing, integrating neuroscience and immunology to elucidate mechanisms, developing pain‑risk prediction models, and advancing targeted, steroid‑sparing management strategies. Such efforts will be essential to optimize precision pain management and improve the overall therapeutic index of cancer immunotherapy.

## Supplementary information

Below is the link to the electronic supplementary material.

ESM 1 (XLSX 266 KB)

ESM 2 (XLSX 25.4 KB)

ESM 3 (XLSX 26.7 KB)

## Data Availability

No datasets were generated or analysed during the current study.
